# Assessment of physicians’ awareness and clinical practice regarding gingival enlargement caused by calcium channel blockers

**DOI:** 10.7717/peerj.20739

**Published:** 2026-02-10

**Authors:** Banaz Jabbar Ali, Al-Hussein Safaa Hussein, Ban Karem Hassan, Ola Shakir Fadhil, Ammar Sh Ahmed

**Affiliations:** 1 Department of Periodontics, College of Dentistry, Mustansiriyah University, Baghdad, Iraq; 2 Department of Clinical Pharmacy, College of Pharmacy, Al-Bayan University, Baghdad, Iraq; 3 Director of Comprehensive Care for Non-Communicable Diseases Section, Public Health Directorate, MoH-Iraq, Baghdad, Iraq; 4 Department of Pedodontics, Orthodontic and Preventive Dentistry, College of Dentistry, Mustansiriyah University, Baghdad, Iraq

**Keywords:** Gingival enlargement, Calcium blockers, Medications, Knowledge, Attitude

## Abstract

**Background:**

Gingival enlargement is a side effect of calcium channel blockers (CCBs), which are usually used to treat cardiovascular conditions. Physicians have an important role in identifying and managing the disease, but their level of awareness and ability to apply their knowledge in clinical practice remain unclear in Iraq.

**Objective:**

This study aims to assess physicians’ knowledge of calcium channel blocker (CCB)-induced gingival enlargement and determine whether they apply this knowledge in their clinical practice.

**Methods:**

A cross-sectional survey was conducted among 331 physicians working in Iraqi primary healthcare institutions to collect the data for this study. A Google Forms questionnaire was designed, created, and distributed *via* Facebook and WhatsApp (special groups for primary health centers in Iraq). The survey evaluated their understanding about gingival enlargement as a side effect of calcium channel blockers.

**Results:**

The study analyzed responses to questions regarding awareness and management of gingival enlargement linked with calcium channel blocker (CCB) drugs. Only 58% of physicians reported that CCB use causes gingival enlargement as a side effect. However, fewer responders (33.5%) believed they informed patients about this risk. Furthermore, 57.7% of respondents said they do not regularly refer patients for gingival assessment related to CCB use. When asked about factors affecting the occurrence and severity of gingival enlargement, the majority (88.2%) identified a combination of individual susceptibility, dental hygiene levels, and specific medication as relevant factors for this. Most respondents (73.4%) approved that improving oral hygiene and professional cleanings assist in managing drug-induced gingival enlargement.

**Conclusion:**

There is a gap between the knowledge of physicians regarding the role of calcium channel blockers in causing gingival enlargement and their clinical practice. Enhancing medical-dental collaboration and providing targeted education can lead to better management and outcomes for patients experiencing CCB-induced gingival enlargement.

## Introduction

Gingival enlargement is a clinical condition characterised by both hyperplasia and hypertrophy of the gingival tissues. Its aetiology is multifactorial, primarily involving chronic inflammation caused by microbial dental plaque, as well as influences from hormonal changes, genetic predisposition, neoplastic conditions (both benign and malignant), and various local and systemic factors, such as drugs (Beaumont et al., 2017).

Drug-induced gingival enlargement (DIGE) has a different therapeutic relevance because the physician who prescribed it can frequently change or replace the medicines that cause it. Four types of medications are most frequently linked to gingival enlargement: immunosuppressants, calcium channel blockers (CCBs), anticonvulsants, and high-dose oral contraceptives. It is hard to tell these medications changes apart because, even though they work differently in the body, the changes they cause in the gingiva look similar both in how they appear and under a microscope (Murakami et al., 2018). Other studies have reported that human gingival enlargement lesions caused by ciclosporin, phenytoin, and nifedipine differ in their cellular and tissue properties, strong evidence indicates that these lesions vary depending on the medication used. A thorough examination of human gingivectomy samples comparing cellular and molecular characteristics showed that lesions caused by phenytoin are notably the most fibrotic, those caused by ciclosporin are quite inflammatory with minimal fibrosis, and those caused by nifedipine are mixed (Uzel et al., 2001).

Among these drug classes, calcium antagonists are medications used to treat cardiovascular issues. They prevent calcium ions from entering the cell membranes of the heart and smooth muscle cells, stopping calcium from moving inside the cells. This leads to the dilation of the coronary arteries and arterioles, improving oxygen supply to the heart muscle and lowering blood pressure by dilating peripheral veins. Gingival hyperplasia may develop as a result of taking some of these medicines (Livada & Shiloah, 2014).

The interaction of the cell membrane with calcium channel blockers (CCBs) involves a complex mechanism, where three distinct yet allosterically connected receptors have been identified. Several classes of chemical compounds target these receptors: (1) Dihydropyridines, such as amlodipine and isradipine, which belong to the nifedipine-like group; (2) phenylalkylamines, such as verapamil and its related drugs; and (3) benzothiazepines, including diltiazem and similar medications. These drug classes were specifically designed to bind to their respective receptor sites. CCBs have been introduced in three successive generations (Toyo-Oka & Nayler, 1996). Developed in the 1970s, the first generation included agents from all three main types: benzothiazepines, phenylalkylamines, and dihydropyridines; however, these early drugs were associated with several side effects, such as rapid heart rate, facial flushing, and gingival enlargement (Dougall & McLay, 1996). Later generations of dihydropyridines—such as nitrendipine (Brown, Sein & Corio, 1990), oxydipine (Nyska et al., 1990), and amlodipine (Jorgensen, 1997)—were created to reduce these side effects. Nonetheless, drug-induced gingival enlargement continued to pose concerns for some patients (Whitworth & Chalmers, 2004).

The first reported case of amlodipine-induced gingival enlargement was published by Seymour et al. (1994). Subsequent reports, such as that by Lafzi, Farahani & Shoja (2006) documented gingival hypertrophy occurring within just two months of starting a daily 10 mg dose of amlodipine. Usually, the enlargement appears within one to three months, beginning at the interdental papillae of the anterior labial surfaces (Malek, El Houari & Kissa, 2019). From painless, beadlike expansion of the interdental papilla, the growth spreads to the lingual and facial gingival areas. The papillary and marginal enlargements merge as the disease worses, sometimes forming a large tissue fold covering significant areas of the crowns (Newman et al., 2006). The enlargement is mulberry-shaped, firm, pale pink, robust, and has a minutely lobulated surface when inflammation is absent. It also tends not to bleed. A linear groove separates the enlargement from the gingival margin, which is typically where it appears to protrude (Newman et al., 2006). Although more prominent in the maxillary and mandibular anterior regions, the enlargement is typically present throughout the mouth. It happens in tooth-containing areas rather than in edentulous spaces, and the expansion goes away in tooth extraction locations. Mucosal enlargement in edentulous mouths has been documented, though it is rare (Newman et al., 2006).

Since inflammation caused by bacterial biofilm acts as a causal factor, the occurrence and extent of plaque accumulation are closely associated with the severity of DIGE (Samudrala et al., 2016). When gingival enlargement is present, effective plaque control becomes more difficult, leading to subsequent inflammatory changes that increase the enlargement. These changes could include bluish discolouration or erythema, loss of gingival contour, and an increased risk of bleeding (Newman et al., 2006).

The degree of plaque accumulation and inflammation produced by plaque was thought to be strongly correlated with the severity of gingival enlargement, which appeared to be a contributing component (Samudrala et al., 2016).

Beyond its clinical complications, DIGE can significantly affect a patient’s quality of life, the enlargement may affect the aesthetic, disrupt mastication and speech, and make maintaining good oral hygiene difficult. These issues raise the risk of dental caries and periodontal disease and could even effect nutritional status (Amit & Shalu, 2012).

The plaque-induced inflammation, genetic influences, and medication variables are the main three factors affect the manifestation of gingival alterations. A complete history and careful clinical examination form represent the base for the DIGE diagnosis. The two primary treatment options for DIGE are non-surgical and surgical procedures, which are generally combined with maintenance therapy. Before starting treatment, it is recommended to stop or alter medications, followed by implementing plaque control (non-surgical procedure). Periodontal surgery should be considered if gingival enlargement continues to impair function or aesthetics. Maintaining oral hygiene and periodontal care throughout treatment is essential (Samudrala et al., 2016; Bán, Pintér & Kun, 2018; Chang et al., 2018; Singh & Singh, 2019).

Despite the clinical significance of DIGE, there is a lack of research on medical physicians’ awareness of drugs that cause gingival enlargement. Early detection and patient education can greatly reduce complications.

Therefore, this study aims to assess the medical physicians’ awareness of link between the drug and gingival enlargement.

In Iraq, primary healthcare centers (PHCs) represent the front line of the healthcare system, and they offer early detection of hypertension by screening all adults above 20 years of age who attend PHCs for any reason. When a patient receives a diagnosis of hypertension, the PHCs open a medical file and provide them with treatment, counseling, and follow-up appointments.

Although physicians at healthcare centres are not qualified to diagnose drug-induced gingival enlargement, they can help raise awareness and provide guidance. These physicians must understand this issue so they can educate patients receiving drugs cause this side effect such as calcium channel blockers about the importance of maintaining good oral health. They should advise patients to organise regular dental check-ups. Furthermore, patients should be warned about the potential for gingival enlargement as a side effect of these medications, which necessitates careful oral care and early detection to prevent future complications.

## Materials & Methods

In this study, 331 questionnaires were collected from specialized physicians working in primary healthcare centers in Iraq. These centers represent the first point of diagnosis and patient care in the country.

The sample size was calculated using G*Power software, based on the expected effect size and statistical power the estimated a minimum participants are 220. However, the sample was increased to 331 to improve result accuracy and enhance statistical representation. This study was approved by the scientific committee at the College of Dentistry, Mustansiriyah University (no. 784). At the beginning of the Google Form, the participants saw a clear statement: “Understanding your insights will help enhance multidisciplinary collaboration, improve patient care, and increase awareness of oral health management in patients on these medications”. Participants indicated their agreement to take part voluntarily by completing the questionnaire after reading this statement.

### Questionnaire design

The authors developed a questionnaire specifically for the study. The questionnaire was initially designed and distributed in English, as English is the official language of instruction, documentation, and professional communication in the medical field in Iraq. A Google Forms questionnaire was created, developed, and shared through Facebook and WhatsApp (special groups for primary health center in Iraq). The main concept for the questionnaire design was based on a similar study (Ugale et al., 2022). The survey was distributed exclusively through an official closed social media group compose of physicians working in primary healthcare centers. The questionnaire was directly sent to these physicians by one of the researchers, only suitable participants (primary care physicians) received the questionnaire, fulfilling the inclusion criteria by default.

The questions were designed to reflect the study’s objectives. The first paragraph gathered general information, such as age, sex, specialization, and years of practice. The second part consisted of six questions.

The questions aimed to explore the physicians’ awareness of the potential for calcium channel blockers to cause gingival enlargement, whether they inform patients about this side effect, and if they refer them for gingival evaluation during treatment. In addition, the questions addressed their views on preventive or management strategies for this condition.

### Statistical analysis

The data were statistically analyzed using IBM SPSS Statistics 30.0.0.0 software. Descriptive statistics were employed to examine demographic factors such as gender, age, and years of experience. The Pearson Chi-square test was applied to analyze the responses given to these questions. The *p*-value of ≤ 0.05 was considered statistically significant.

## Results

Table 1 represented the descriptive statistics, including age, gender and years of clinical practice.

In regard to calcium channel blocker (CCB)-induced gingival enlargement (GE), Table 2 displays the physicians’ answers to awareness and practice-related questions, including the percentage and number of responses for each choice.

As regards whether (the consumption of CCB can cause gingival enlargement? “Yes” was the response given by 58% of physicians, revealing a moderate level of awareness about this side effect.

The distribution of responses about the question (Do you inform patients who are prescribed CCBs about the possible risk of gingival enlargement?) was as follows: No: 46.5%, Maybe: 19.9%, and Yes: 33.5%.

“Do you routinely refer patients on CCBs to check their gingival status?” was the question asked. Only 18.4% reported routine referrals, compared to 57.7% who said “no”.

Physicians were also asked about the factors that contribute to the incidence and severity of gingival enlargement. Most respondents (88.2%) indicated that all these factors play a role in the condition. The responses for each option were as follows: individual susceptibility (4.2%), oral hygiene status (4.8%), and specific medication (2.7%).

**Table 1:** Demographic characteristics of the study sample.

**Variables**		**Frequency**	**%**
**Gender**	Male	111	33.5
	Female	220	66.5
**Age**	25–34	132	39.9
	35–44	90	27.2
	45–54	59	17.8
	55–64	43	13
	65 and above	7	2.1
**Specialization**	Family medicine	140	42.3
	Others	191	57.7
**Years of experience**	0–5	94	28.4
	6–10	70	21.1
	11–20	78	23.6
	21–30	59	17.8
	31 and above	30	9.1

**Notes.**

Percent (%).

**Table 2:** Participants’ responses to the questions.

**Question**	**Response**	**Frequency**	**%**
Q1- Does intake of Calcium Channel Blocker drugs induce gingival overgrowth as a side effect?	Yes	192	58
	No	37	11.2
	Maybe	102	30.8
Q2- Do you inform patients prescribed Calcium Channel Blocker medications about the potential risk of gingival overgrowth?	Yes	111	33.5
	No	154	46.5
	Maybe	66	19.9
Q3- Do you routinely refer patients on Calcium Channel blockers to check their gingival status?	Yes	61	18.4
	No	191	57.7
	Maybe	79	23.9
Q4- The overall incidence and severity of gingival overgrowth depend on	Individual susceptibility,	14	4.2
	Oral hygiene status	16	4.8
	The specific medication	9	2.7
	All the above	292	88.2
Q5- Are you aware of the potential benefits of improving oral hygiene and professional dental cleanings in managing drug-induced gingival overgrowth?	Yes	243	73.4
	No	37	11.2
	Maybe	51	15.4
Q6- Line of treatment for drug-induced gingival overgrowth	Switching to alternative medications	169	51.1
	Surgical excision	3	9
	Both of them	159	48

**Notes.**

Percent (%).

When asked whether the potential benefits of improving oral hygiene and professional dental cleanings in managing drug-induced gingival, most physicians (73.4%) responded “Yes”, indicating good awareness of the role of oral health in controlling the condition.

For the question regarding the line of treatment for drug-induced gingival enlargement, the responses were as follows: switching to alternative medications (51.1%), surgical excision (9%), and both of them (48%).

Table 3 shows that only 50.5% of doctors who reported knowing that calcium channel blockers (CCBs) could cause gingival enlargement also said they often inform their patients about this potential side effect. Furthermore, just 27.6% of them stated they refer patients for a periodontal assessment. Despite limited clinical activity, 90.1% of respondents recognized that the occurrence and severity of gingival enlargement depend on various factors, such as the medication used, oral hygiene, and individual susceptibility. Additionally, 77.6% acknowledged the benefits of improved oral hygiene and professional dental cleanings in managing drug-induced gingival enlargement. Regarding the most suitable treatment approach, 53.1% of respondents indicated that a combination of surgical excision and switching to different medications was the preferred method.

**Table 3:** Distribution of physicians to questions (2–6) based on their awareness of whether intake of calcium channel blocker drugs induce gingival overgrowth as a side effect? (Q1).

**Question**	**Response**	**Yes**	**No**	**Maybe**	***p*-value**
**Q2-** Do you inform patients prescribed Calcium Channel Blocker medications about the potential risk of gingival overgrowth?	Yes	97	4	10	<.001
		50.50%	10.80%	9.80%	
	No	51	30	73	
		26.60%	81.10%	71.60%	
	Maybe	44	3	19	
		22.90%	8.10%	18.60%	
**Q3-** Do you routinely refer patients on Calcium Channel blockers to check their gingival status?	Yes	53	3	5	<.001
		27.60%	8.10%	4.90%	
	No	78	27	86	
		40.60%	73.00%	84.30%	
	Maybe	61	7	11	
		31.80%	18.90%	10.80%	
**Q4-** The overall incidence and severity of gingival overgrowth depend on	Individual susceptibility,	5	3	6	0.005
		2.60%	8.10%	5.90%	
	Oral hygiene status	8	6	2	
		4.20%	16.20%	2.00%	
	The specific medication	6	2	1	
		3.10%	5.40%	1.00%	
	All the above	173	26	93	
		90.10%	70.30%	91.20%	
**Q5-** Are you aware of the potential benefits of improving oral hygiene and professional dental cleanings in managing drug-induced gingival overgrowth?	Yes	149	28	66	0.188
		77.60%	75.70%	64.70%	
	No	18	3	16	
		9.40%	8.10%	15.70%	
	Maybe	25	6	20	
		13.00%	16.20%	19.60%	
Q6- Line of treatment for drug-induced gingival overgrowth	Switching to alternative medications	90	22	57	0.006
		46.90%	59.50%	55.90%	
	Surgical excision	0	2	1	
		0.00%	5.40%	1.00%	
	Both of them	102	13	44	
		53.10%	35.10%	43.10%	

**Notes.**

Percent (%).

Awareness level is based on responses to Question 1.

**Figure 1:**
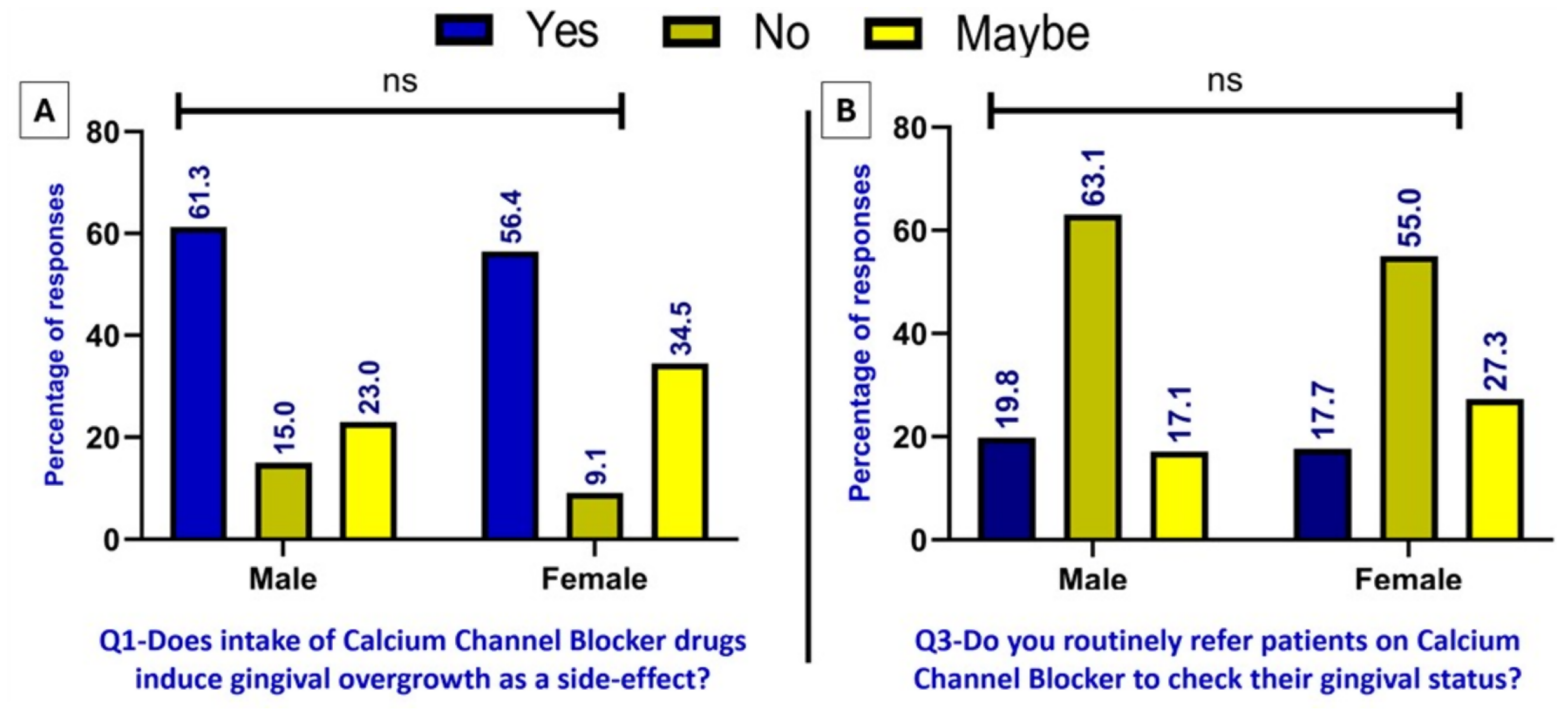
(A–B) Awareness *vs.* application: gender differences in knowledge and referral of CCB patients for gingival evaluation (significant at * *p* < 0.05, ** *p* < 0.01, *** *p* < 0.001 using chi-squared test).

Figure 1A shows the percentage of male and female respondents who answered the first question, Q1 (Does intake of calcium channel blocker drugs cause gingival enlargement as a side effect?). This demonstrates which group received a higher ‘yes’ response. Meanwhile, Fig. 1B indicates that although over half of the respondents answered ‘Yes’ to the first question, the number of ‘Yes’ responses to the third question, “Do you routinely refer patients on calcium channel blockers to check their gingival status?” was notably lower. It found no significant link between gender and the answers to the questions.

**Figure 2:**
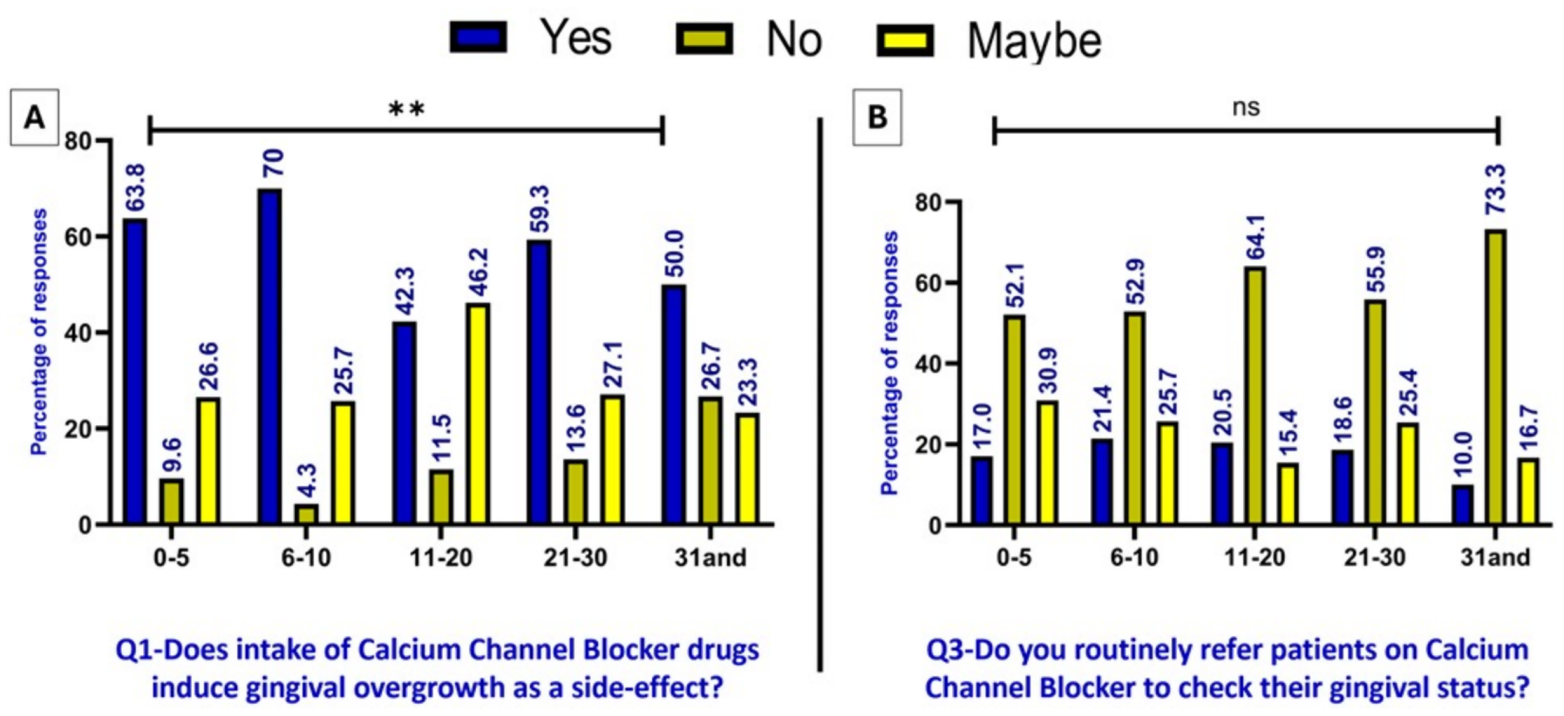
(A–B) Awareness *vs.* application: differences in referral practices for CCB patients across experience levels (significant at * *p* < 0.05, ** *p* < 0.01, *** *p* < 0.001 using chi-squared test).

Figure 2A displays the distribution of responses to Q1 (Does intake of calcium channel blocker drugs cause gingival enlargement as a side effect?), based on years of experience, which reveals a significant association between the years of experience and the response. Meanwhile, Fig. 2B shows that although the majority answered ‘Yes’ to the first question, the ‘Yes’ responses were noticeably fewer when asked about Q3—Do you routinely refer patients on calcium channel blockers to check their gingival status? “No significant association was found between the percentage of responses and the participants’ years of experience.

## Discussion

Studies have shown that certain drugs, such as calcium channel blockers, anticonvulsants, and immunosuppressants, can induce gingival enlargement (DIGE). Despite its clinical relevance, there is a significant gap in awareness and management across primary healthcare facilities. In an Ethiopian study that assessed healthcare workers’ awareness of medication side effects, 66% of participants said they were aware of them, and 49.1% reported having personally experienced one (Seid et al., 2018). Another study on the side effects of medications among Vietnamese physicians, nurses, and pharmacists found that only 59.3% had reported this condition at least once in their careers, and 56.2% said they could recognise the side drug interactions (Le et al., 2020). Additionally, a Turkish study revealed that although physicians are somewhat aware of the drugs that cause gingival enlargement, their knowledge remains insufficient (Çetin Özdemir & Uzunkaya, 2024).

While 58% of physicians in this study agreed that CCBs are associated with gingival enlargement, only 33.5% advised patients about this risk, and just 18.4% referred their patients for a gingival assessment. These findings are consistent with earlier studies (Ugale et al., 2022), in which only 21.56% of physicians referred these patients to dentists.

The situation highlights a substantial gap between what physicians know and what they do, as well as unclear primary care guidelines and a lack of importance on the relationship between dental health and general health in medical education.

The results presented that over half of the people surveyed (58%) were aware that calcium channel blockers might be linked to gingival enlargement, and 73.4% understood that better oral hygiene and professional dental cleanings could help manage this problem; however, 46.5% did not regularly inform patients about this side effect, Furthermore, only 18.4% reported recommending patients for examining the gingival status, indicating a gap between awareness and clinical practice.

This is similar to the findings of a previous study (Ugale et al., 2022). Among all the participants, only 21.56% referred these patients to dental practitioners.

A total of 73.4% of respondents agreed that receiving regular professional dental cleanings and maintaining good oral hygiene could help reduce DIGE, This finding supports studies showing how plaque-induced inflammation degrades the drug-induced gingival enlargement (Seymour, Thomason & Ellis, 1996).

A total of 88.2% some participants acknowledged that various factors, including dental hygiene, personal susceptibility, and the type of drug, contribute to the disease. Research has shown that the body-environment relationship plays a significant role in the development of drug-induced gingival enlargement (DIGE).

Notably, 48% of physicians selected a combination of medication modification and surgery, even though 51.1% preferred changing the drug as the primary treatment. Just 9% favorted surgery alone. Current guidelines are reflected in these opinions. Consistent professional prophylaxis should be maintained during drug administration. Clinical signs and relapse often appear four weeks after drug change and consistent oral hygiene. However, if symptoms persist over a long period, only partial regression may occur. If medication substitution is not an option or minimal regression is observed despite the adjustment, gingival remodelling may be necessary (Feijó Miguelis et al., 2019).

Therefore, in these situations, surgical intervention takes priority, with scalpel gingivectomy continuing to be the recommended course of treatment (Mavrogiannis et al., 2006b; Mavrogiannis et al., 2006a).

The best course of action for treating drug-induced gingival enlargement is to stop taking the drug or switch it out. Gingival lesions usually go away in one to eight weeks after using this technique (Khocht & Schneider, 1997).

Unfortunately, not all patients respond well to this treatment, particularly those with long-standing or chronic gingival lesions (Marshall & Bartold, 1999).

Nevertheless, evidence suggests that nonsurgical periodontal therapy can reduce gingival tissue inflammation, which can decrease the need for surgery (Somacarrera et al., 1997).

Conservative nonsurgical periodontal therapy, including frequent professional prophylaxis and a home care regimen, can be followed. Reports also suggest that patients completing a vigorous oral cleanliness program may see a retreat in gingival hypertrophy over time (Montebugnoli, Servidio & Bernardi, 2000).

The results shown in Table 3 demonstrate a significant discrepancy between the clinical practices of physicians and their knowledge of calcium channel blocker-induced gingival enlargement (CCB-DIGE). While 90% of the physicians answered yes to the question (Does intake of Calcium Channel Blocker drugs induce gingival enlargement as a side effect?), although they were able to accurately identify the multifactorial aetiology of gingival enlargement, which includes medication type, oral hygiene status, and individual susceptibility, only 50.5% of them reported regularly informing patients about this possible side effect. This gap indicates that many physicians might neglect the practical importance of oral health education or neglect to give it top priority.

Despite the known importance of dental evaluation in the treatment of DIGE, only 27.6% of them advised patients about regular checks of gingival health. A total of 77.6% of participants recognized the importance of routine dental cleanings and better oral hygiene in managing DIGE. Furthermore, 53.1% of them believed that a combination of surgical excision and switching to an alternative medication was the best course of treatment.

The results of this study focus on the gap between awareness and clinical practice regarding drug-induced gingival enlargement, particularly regarding calcium channel blockers (CCBs). Although many physicians have knowledge of the importance of managing gingival enlargement, practical barriers such as limited time during patient consultations, the absence of referral systems, and competing medical priorities can limit their ability to properly inform patients and refer them to dental professionals. The approaches of treatment, from medication adjustments to surgical involvements, confirm the need for standardized guidelines.

To enhance patient outcomes, it is critical to bridge this gap by developing better communication between primary care centers and dental professionals. This collaboration, combined with improved physician training and clear referral protocols, can help prevent severe cases and enable timely intervention. When evaluating the findings’ relevance to different healthcare systems or global contexts, care should be taken in how they are understood. Additionally, we suggest that future multinational research investigate physicians’ knowledge and practices around DIGE in larger, more diverse settings.

## Conclusions

Despite of the knowledge of physicians about the link between calcium channel blockers and gingival enlargement, routine assessment of gingival health during follow-up visits and referrals to dental specialists remain limited.

Enhancing awareness through combined dental-medical educational programmes can help address this gap, which is crucial to ensure that patients on these medications are properly referred to dental clinics. Furthermore, emphasising the importance of maintaining optimal oral hygiene should be a key part of the treatment plan, as it is vital for both the prevention and management of this condition.

## Limitations

This study, conducted in collaboration with PHC physicians working at the Ministry of Health, provides important new information about physicians at primary healthcare center in Iraq and their awareness of drug-induced gingival enlargement. Comparisons among different medical specialities working in primary healthcare centres were not possible due to the small sample size, which limited understanding of the importance of collaboration between specialists and dentists. The researchers restricted the questionnaire to six closed-ended items to encourage participation and emphasise the importance of dental monitoring during medication therapy. Data were collected from physicians within an official primary healthcare centres group, where view statistics were inaccessible, so the response rate could not be determined, which may slightly limit generalisability.

## Supplemental Information

10.7717/peerj.20739/supp-1 Supplemental Information 1 Drugs induced gingival enlargement data

10.7717/peerj.20739/supp-2 Supplemental Information 2 Questionnaire

## Additional Information and Declarations

### Data Availability

The following information was supplied regarding data availability: The raw data are available in the Supplemental File.
