# Supplementary material for: Assessment of physicians’ awareness and clinical practice regarding gingival enlargement caused by calcium channel blockers
Source: PeerJ. 2026 Feb 10;14:e20739. doi: 10.7717/peerj.20739 (PMC12903892; doi:10.7717/peerj.20739)
Supplement: Supplemental Information 2 [file peerj-14-20739-s002.docx]

**Assessment of Awareness among Physicians Regarding Gingival Overgrowth Induced by Calcium Channel Blocker**

)Understanding your insights will help enhance multidisciplinary collaboration, improve patient care, and increase awareness of oral health management in patients on these medications(

- **Gender**
  - Male
  - Female
- **Age**
  - 25-34
  - 35-44
  - 45-54
  - 55-64
  - 65 and above
- **Specialization _______________________________**
- **Years of Experience**
  - 0-5
  - 6-10
  - 11-20
  - 21-30
  - 31and above

**Q1- Does intake of Calcium Channel Blocker drugs induce gingival overgrowth as a side-effect?**

- - Yes
  - No
  - May be

**Q2- Do you inform patients prescribed Calcium Channel Blocker medications about the potential risk of gingival overgrowth?**

- - Yes
  - No
  - May be

**Q3-Do you routinely refer patients on Calcium Channel Blockers to check their gingival status?**

- - Yes
  - No
  - May be

**Q4-The overall incidence and severity of gingival overgrowth depend on**

- - Individual susceptibility,
  - Oral hygiene status
  - The specific medication
  - All the above

**Q5-Are you aware of the potential benefits of improving oral hygiene and professional dental cleanings in managing drug-induced gingival overgrowth?**

- - Yes
  - No
  - May be

**Q6-Line of treatment for drug-induced gingival overgrowth**

- - switching to alternative medications
  - Surgical excision
  - Both of them

[(https://docs.google.com/forms/d/e/1FAIpQLSfv4LM4a_ws58GIDIasKrGp2dwNk3RWXah4NPraInAsdGWvcA/viewform?usp=header)](file:///C:\Users\Lenovo\Desktop\حسين%20وعلا22222\(https:\docs.google.com\forms\d\e\1FAIpQLSfv4LM4a_ws58GIDIasKrGp2dwNk3RWXah4NPraInAsdGWvcA\viewform%3fusp=header))
